# Mental health promotion knowledge and practices of nursing students: A qualitative study

**DOI:** 10.4102/hsag.v31i0.3530

**Published:** 2026-10-09

**Authors:** Regomoditswe C. Legwabe, Neltjie C. van Wyk, Varshika M. Bhana-Pema

**Affiliations:** 1 Department of Nursing Science, Faculty of Health Sciences, University of Pretoria, Pretoria, South Africa

**Keywords:** mental health promotion, nursing students, students’ support services, Parental support, stressfull events

## Abstract

**Background:**

High levels of mental distress have been documented among nursing students, but only a few seek help to promote or manage their mental health. Although health promotion is included in their curricula and institutional mental health services are provided to support them in managing the stress they experience during their training, it appears as if the students do not practice mental health promotion for themselves.

**Aim:**

The study aimed to explore and describe nursing students’ personal knowledge and the systemic barriers to mental health promotion.

**Setting:**

The research was done at a nursing education institution in the Gauteng Province of South Africa.

**Methods:**

An exploratory, descriptive contextual qualitative study was conducted with eleven undergraduate students during the 2024–2025 academic year. Data were collected through individual, semi-structured face-to-face interviews until saturation was reached, followed by a thematic analysis.

**Results:**

Three categories were identified, namely ‘rely on self, family and friends,’ ‘depend on lecturers and institutional support services’ and ‘the need for mental health promotion for students’.

**Conclusion:**

Nursing students at the institution were partially knowledgeable about mental health promotion but not all practised appropriate health-promoting mechanisms when dealing with academic and personal challenges.

**Contribution:**

Nursing students need to be supported in their academic journey to become professional nurses. The authors recommend that institutions should adopt mental health-supportive campus cultures to improve students’ mental health, encourage the maintenance of peer support groups and initiate extracurricular health-promoting activities.

## Introduction

Students who are usually new to tertiary education may experience stressful circumstances associated with young adulthood and may be exposed to events that cause mental health challenges during theoretical and practical nursing training (McDermott et al. 2020:11). Many may be expected to manage potentially difficult situations involving patient care during experiential learning (Pascoe et al. 2021:3). Such challenges may contribute to the occurrence of students’ maladaptive coping skills, episodes of sleep deprivation, bad lifestyle habits and incidents of substance misuse, which can exacerbate pre-existing mental health challenges (Panadero, Fraile & García-Pérez 2020:3). Stressful events may also negatively impact students’ performance, leading to poor assessment outcomes (Sapthiang, Van Gordon& Shonin 2019:112–119).

According to Devakani et al. (2019:47), nursing students in India often complain of stressful situations that they find difficult to manage. The findings of a study of Iranian nursing students revealed that they often experienced sleep difficulties, signs of anxiety and psychosomatic complaints (Mohebbi et al. 2019:4). Similar results were obtained in a study of nursing students in the United States of America who, according to Zhang, Peters and Chen (2018:6), displayed stress-related conditions such as migraines and incidents of severe anxiety. A study in the Eastern Cape province of South Africa indicated that nursing students regularly complain about too many demands and too little help from lecturers to meet the objectives of their theoretical and practical training (Stubin, Ruth-Sahd & Dahan 2024:119, 120). Training to become professional nurses is challenging, and according to Albaqawi (2018:109), students often have little time for physical exercise and sleep while they tend to use chemical substances to boost low energy levels.

Mental health promotion seeks to foster individual competencies and to enable communities to prevent mental disorders and enhance quality of life for people (Savitsky et al. 2020:12). Nursing education institutions in South Africa offer support to enable students to prevent and manage their mental health challenges. Students are offered support to increase their competence in problem solving and to enhance their general well-being (Fusar-Poli et al. 2021:200). Despite available support services, little is known about how nursing students translate mental health knowledge into personal practice within the South African nursing education context. According to the first author’s experience as a lecturer at the designated institution, students do not optimally use the institutional health-promoting services. The study therefore aimed to explore and describe nursing students’ personal knowledge and the systemic barriers to mental health promotion at the designated institution.

## Research methods and design

### Research setting

The research was done in the Gauteng province of South Africa, on a campus of a designated nursing education institution. At the time of the study, the campus had 75 lecturers and seven professional nurses involved in clinical training, with 106 support staff members. The campus enrolled 50 students per year. The student counselling department offered counselling for individuals and groups, academic support such as the development of effective study methods, improvement of mental and general health and referral to multidisciplinary healthcare teams. At the time of the research, the department was staffed by a departmental head and two lecturers with master’s degrees in nursing education and certificates in counselling. Utilisation of the services varied during the academic year with a peak period during examinations.

### Design

An exploratory, descriptive and contextual qualitative research approach was used (Polit & Beck 2020:200). The participants’ knowledge and practices of mental health promotion were explored and described as they appeared in the context of the designated nursing education institution. Through qualitative research, the phenomenon was studied in-depth to appreciate its complexity. According to Burns and Grove (2020:150), qualitative research is associated with subjective approaches to thoroughly understand phenomena as they occur in a natural environment. As the researchers conducted an exploratory study, an inductive approach was chosen to allow themes to emerge from participants’ experiences without being constrained by predefined constructs.

### Study population and sampling

The study population consisted of 426 students (140 in level 1; 139 in level 2; and 147 in level 3) enrolled in a 3-year diploma nursing programme during the 2024–2025 academic year. The inclusion criteria specified that all students registered for the programme could be purposively sampled. The first author discussed the proposal with the guardian lecturers of each year group of students and arranged with them to address the students about the study. During the interaction with students, the researcher invited students who were interested in taking part in the study. Appointments with them were arranged during which they could read the study’s participant information leaflet, ask questions about uncertainties and finally decide whether they wanted to participate. The sample size was determined by data saturation and included 11 participants aged 22–28 years. The authors agreed that the interviews with participants 10 and 11 did not provide new information.

### Data collection

The first author collected the data through individual semi-structured face-to-face interviews in English at venues within the institution preferred by the participants. All participants agreed to and gave written permission for the audio-recording of the interviews. Audio-recording of the interviews enabled the researcher to concentrate on their content instead of focusing on writing complete records. Field notes were also used to record data that could not be recorded, such as non-verbal communication through gestures and emotional reactions. Non-verbal gestures play a crucial role in research interviews as they often convey information beyond what words alone can express (Creswell & Creswell 2018:59). The interview guide addressed the participants’ understanding of mental health promotion, the ways in which they managed stressful events, their use of the services delivered by the institutional counselling department and the activities that they wished the institution could implement to improve their mental health. The interviews were no longer than 45 min and continued until data saturation was obtained.

### Data analysis

A thematic data analysis was done. Harding (2019) describes thematic data analysis as inductive, implying that it moves from the particular to the general. The first author conducted the analysis following the guidelines of Braun and Clarke (2023), and the second author co-coded the data to ensure that the categories and sub-categories correspond with the participants’ input. After completing the transcription of the recorded interviews and typing the field notes, the data were thoroughly studied, enabling the researchers to become familiar with the content. Phrases with similar content were identified and grouped into sub-categories that were later grouped into categories. As thematic analysis is not a linear process, the researchers repeatedly compared the categories and sub-categories with the raw data to ensure that they fit the content of the transcribed interviews.

### Trustworthiness

The use of both interviews and field notes enabled the researchers to enhance the truth value of the findings. The first author also asked four participants whether they were satisfied that the findings reflected the data they had shared with her to contribute to the credibility of the research (Harding 2019). The applicability of the findings was ensured through the strategy of transferability (Polit & Beck 2018). The strategy refers to whether the findings of one study can be applied to similar contexts. The researchers comprehensively described the study’s context and the methodology of the research, enabling others to decide whether the findings are transferable to similar situations. The dependability of the findings was ensured through the involvement of the second author as a co-coder of the data. The first and second authors coded the interview transcripts and field notes and compared and debated the outcome until they agreed that the final set of categories and sub-categories reflected the input of the participants. The first author also kept a reflexive journal to note possible ways in which she could have influenced the findings during data collection and analysis to ensure the neutrality of the findings. The neutrality of the findings is, according to Burns and Grove (2020), protected when researchers do their utmost best not to influence the outcome of the research. The study adhered to the Consolidated Criteria for Reporting Qualitative Research guidelines to ensure quality research and findings.

### Ethical considerations

Ethical clearance to conduct this study was obtained from the Research Ethics Committee of the Faculty of Health Sciences of the University of Pretoria (No. 269/2024). Additionally, the applicable authorities permitted the research to be conducted at the designated institution (Reference number: GP 202407062). The proposal was comprehensively discussed with the prospective participants, and only those who voluntarily provided written informed consent were included in the study. The confidentiality of the participants was ensured, and only codes were used in the research reports to protect their privacy. The participants’ names were not mentioned, and the information that they shared with the researchers was presented in such a way that it could not be associated with specific participants. The first author discussed her position as a lecturer at the designated institution and assured the participants that no personal and sensitive information shared with her would be shared with other lecturers and the management of the institution.

## Results

Eleven participants were interviewed. Three of them were male students, and eight were female students. Three students were in Level 1, five in Level 2 and three in Level 3 of the undergraduate nursing programme. The demographic profile summary is presented in Table 1.

**TABLE 1:** Demographic data of participants.

Participants	Age	Gender	Level of study
P1	26	Female	Level 2
P2	25	Female	Level 2
P3	23	Female	Level 2
P4	24	Female	Level 2
P5	26	Male	Level 3
P6	26	Female	Level 1
P7	22	Female	Level 2
P8	25	Female	Level 1
P9	26	Female	Level 1
P10	27	Male	Level 3
P11	28	Male	Level 3

### Categories and sub-categories

Three categories and nine sub-categories were identified. Refer to Table 2.

**TABLE 2:** Categories and sub-categories.

Categories	Sub-categories
Category 1: Rely on self, family and friends	1.1 Keep challenges to self 1.2 Rely on self-care 1.3 Depend on family and friends
Category 2: Depend on lecturers and institutional support services	2.1 Accept support from lecturers 2.2 Explore the institutional mental health promotion service-Student Counselling Department 2.3 Use the Student Representative Council mental health support
Category 3: The need for mental health promotion for students	3.1 Students’ hardships without mental health promotion 3.2 Students thrive with mental health promotion 3.3 Comprehension of the advantages of mental health promotion

#### Category 1: Rely on self, family and friends

The participants commonly stated that they relied on themselves, family and peers as a way to promote their mental well-being. Although they were exposed to numerous challenges, they often preferred to rely on themselves to manage stressful situations:

‘I can’t really talk with people outside and say, I’m struggling. I have to be alone … When I am alone I get time to think and analyse things, and it allows me to take informed decisions.’ (Participant 3, 23, Female)

‘I just try to do whatever I can on my own.’ (Participant 1, 26, Female)

‘I do not want to talk to others … I would rather cry in a corner or cry alone.’ (Participant 6, 26, Female)

Participants indicated that they relied on self-care activities such as praying, going to church and reading the bible to relieve their stress:

‘For me being at church, praise and worshipping brings joy to my heart and after a visit to the church I feel revived and my stress decreased.’ (Participant 3, 23, Female)

‘Prayer session helps me to elevate my mood and helps me relax and let God take control in every situation.’ (Participant 6, 26, Female)

Some participants revealed that they relied on sufficient rest, relaxation and mindfulness or meditation as forms of self-care to promote their mental well-being:

‘I highly recommend relaxation, meditation and rest because it helps your mind to reset, this improves your mental clarity and reduces stress.’ (Participant 5, 26, Male)

‘Leisure means spending time away from school, relaxing alone or being with my friends. I also meditate. Meditation helps me a lot.’ (Participant 6, 26, Female)

Reading and writing were used as forms of self-care by some participants. It helped them to enhance their well-being and to manage stressful events:

‘Lately for me I’ll just write my feelings and thoughts on my notes pad or diary … Rather than bottle my feelings inside.’ (Participant 3, 23, Female)

‘I just read motivational books. Reading helps the body and mind to relax and to think logically especially when overwhelmed.’ (Participant 9, 26, Female)

Some participants engaged in outdoor activities as forms of self-care. They tried to take part in activities as often as possible to promote their mental health. Being active and taking part in sport helped them to cope with challenges associated with being students:

‘I jog at the park, because jogging promotes mental and physical strength and endurance.’ (Participant 6, 26, Female)

‘I feel energetic when I play netball. It helps to relieve any stress I might be having and also improves my mood.’ (Participant 7, 22, Female)

Singing in a choir and listening to music were mentioned as activities that the participants enjoyed. Dancing helped them to loosen up and to cope with stressful events while music healed their minds:

‘Being in a choir is refreshing, singing your lungs out helps to relieve stress.’ (Participant 5, 26, Male)

‘Dancing makes me happy, it also helps to reduce stress and improves my mood.’ (Participant 11, 28, Male)

Turning to family was a common strategy that is often used when the participants were faced with challenges. They indicated that they relied on close family members for support:

‘My parents are helpful in making me manage the stress that comes with being a nursing student.’ (Participant 3, 23, Female)

‘My family forms part of my support structure because they are always available and willing to listen when I need them.’ (Participant 4, 24, Female)

Participants indicated that they often relied on their trusted friends and classmates for mental health support:

‘I usually get a different perspective about things after talking to a friend. It also helps me with self-introspection. Friends also make you realise that you’re not alone in whatever battle you may be facing.’ (Participant 6, 26, Female)

Friends who were also classmates understood the stressful situations that they experienced:

‘The main support is from friends who are my classmates as talking to them relieves the pressure. We are in the same boat.’ (Participant 2, 25, Female)

#### Category 2: Depend on lecturers and institutional support services

The participants relied on their lecturers and the staff of the institutional support services to help them to promote their mental well-being. Lecturers were able to provide support when they experienced academic and personal challenges: They had regular contact with the participants, who viewed them as sources of support:

‘I would say I experienced the lecturers as supportive … they made me feel that I was not alone in this journey.’ (Participant 3, 23, Female)

They had regular contact with the participants who viewed them as sources of support:

‘With regards to lecturers, that’s where I get the most support because I think that’s the closest people we know and they understand us better.’ (Participant 6, 26, Female)

‘Lecturers are available … their doors are always open.’ (Participant 9, 26, Female)

The participants were aware of the services that the institutional counselling department rendered to students but not many of them made use of it:

‘To my knowledge student counselling supports students who are struggling academically by teaching them study strategies.’ (Participant 6, 26, Female)

‘They also have counselling services where you can go to talk to them. I guess some people go there.’ (Participant 5, 26, Male)

The participants indicated that the Student Representative Council successfully implemented activities that promoted students’ mental well-being:

‘The support from them is effective. It helps us to manage our studies with ease to avoid being stressed out.’ (Participant 7, 22, Female)

‘The availability of the Student Representative Council to assist us on academic matters is highly effective as it promotes a feeling of belonging.’ (Participant 8, 25, Female)

Participants indicated how sport provided within the institution promoted their mental well-being. The Student Representative Council introduced soccer and netball to the students:

‘Sports is effective in improving mood, reducing stress and reducing feelings of loneliness.’ (Participant 3, 23, Female)

According to the participants, taking part in sport improved their mood and reduced feelings of loneliness:

‘I feel energetic when I play netball. This in turn helps to relieve any stress I might be having and also improves my mood.’ (Participant 7, 22, Female)

#### Category 3: The need for mental health promotion for students

Participants emphasised the need for mental health promotion to enable students’ academic achievements and support their general well-being. The absence of institutional mental health promotion activities may lead to students experiencing high levels of distress and poor academic performance. Students with poor mental health may resort to inappropriate behaviour to manage overwhelming challenges:

‘Other students may abuse alcohol to try to cope with academic and personal stresses.’ (Participant 5, 26, Male)

‘The inability to balance school and private life will further cause emotional distress, depression and anxiety to students.’ (Participant 4, 24, Female)

‘Students are going through a lot in such a way that some are even tried to commit suicide.’ (Participant 2, 25, Female)

Institutional efforts to promote students’ mental health are of utmost importance to enable them to develop emotional resilience to thrive in an increasingly complex healthcare world:

‘Most of all, I think mental health promotion will assist us to be able to cope well with everything that’s going on, personal issues and academic challenges.’ (Participant 1, 26, Female)

Participants revealed that promoting mental health within the institution may make them flourish academically and personally. They believed that with institutional support they would develop healthy coping mechanisms:

‘I think it will help prevent students from making wrong decisions. A lot of students are doing things that are not good, thinking it’s the best way to cope.’ (Participant 2, 25, Female)

‘If our mental health is promoted we will come up with better coping skills. We won’t bunk class due to exhaustion but we will be able to balance our private and academic lives.’ (Participant 5, 26, Male)

Institutions should enable students through mental health promotion activities to solve their personal problems and to succeed academically:

‘Students will not struggle with their studies and they will be able to solve their personal problems.’ (Participant 8, 25, Female)

‘If mental health promotion is there, it will help to decrease our stress.’ (Participant 9, 26, Female)

Participants indicated that they will thrive if extracurricular activities are promoted as part of mental health promotion within the institution:

‘Maybe having de-stress days, maybe games before exams, or doing fun activities on campus so that everyone can just de-stress.’ (Participant 8, 25, Female)

‘Time to unwind, assist in removing or offloading stress.’ (Participant 11, 28, Male)

The participants emphasised that students should be made aware of the benefits of taking part in mental health promotion. Mental health is an essential element of complete well-being as it reflects the emotional state and cognitive abilities of an individual and contributes to a meaningful, stable and productive life:

‘I think mental health promotion is raising awareness about mental health issues such as depression and anxiety.’ (Participant 1, 26, Female)

By making use of mental health promotion, students may prevent or timeously respond to illnesses such as depression:

‘The first thing you have to do, is to acknowledge, and accept, what you are going though, and understand that it will be okay. Allow yourself to go through the whole process. Avoid deviating, as you will be running away from the main problem.’ (Participant 3, 23, Female)

In mental health promotion, students are warned not to associate with fellow students who practice unhealthy ways to cope with stressful events:

‘Mental health promotion is promoting your mental well-being, reducing your stress, trying not to have depression and all those by changing your own environment, Or the people that you hang with. It’s like finding support regarding your mental health, especially when you feel like you are not okay.’ (Participant 4, 24, Female)

## Discussion

According to Li et al. (2025:7), nursing students face higher levels of mental health challenges compared to other student populations as they have to manage the demands of classroom instruction and unpredictable clinical situations. Often, an overload of academic work is experienced, and they fear that they may cause medical errors or harm to patients (Buriro et al. 2024:1839). In the research findings, participants revealed that although mental health support was available within the institution, they preferred to keep or solve their challenges by themselves. The findings are in line with the perceptions of Khunsawat et al. (2025:658), who indicate that, based on the stigma surrounding the use of mental health services, students prefer not to seek help, resulting in them keeping their challenges to themselves. Students are also quick to offer reasons for the non-use of counselling services as they believe that they can solve their own problems without relying on professional persons (Huenergarde 2018:42).

According to Cabahug et al. (2025:20), self-care is any activity that people do consciously to take care of their well-being. In the context of nursing students, self-care takes on special relevance, as these future healthcare professionals are expected to be able to care for themselves in order to effectively care for others (Ehsan et al. 2024:5). Participants in this research revealed that they went to church, prayed, read the bible and trusted God to help them manage stressful situations and to prevent mental challenges. The participants further indicated that prayer lifted their mood when they were sad and when things did not go their way. Thus, going to church was their coping mechanism. De Almeida et al. (2020:931) concur with the findings, indicating that students who incorporate religious practice and cultural activities often experience purpose-driven lives. Spiritually grounded students tend to find creative ways to reduce the stress associated with being nursing students. According to Laposha and Smallfield (2022:3), students reported reduced levels of stress and improved health and well-being when they relied on spiritual (‘trusting God’) rather than on scientific (using social networks) sources of support.

The research findings revealed that participants relied on sufficient rest, relaxation and mindfulness or meditation as forms of self-care to promote their mental well-being and to manage stressful events. One of the participants tried to spend sufficient time away from the institution and her study material, as it helped her to feel refreshed. Time spent away from usual responsibilities with the intention of rejuvenating one’s own mental health may reduce episodes of burnout and enhance mental well-being. Gois et al. (2025:1) further indicated that sufficient sleep and rest may not only play a vital role in physical recovery but are also fundamental to enhancing cognitive functioning required for students’ academic achievement. Mindfulness-based interventions may also assist students in coping with stressful events associated with examinations. It may reduce feelings of depression and hopelessness (Kim & Yoo 2025:75).

The findings revealed that some participants engaged in outdoor activities as a form of self-care. They tried to take part in physical activities as often as possible to promote their mental health. Being active and taking part in sport helped them to cope with challenges associated with being students. Zwart and Ewert (2022:873) concur with the above finding, indicating that being in nature enhances physical and psychological health. Taking part in outdoor activities is associated with reduced levels of anxiety and depression, as well as increased self-confidence and life satisfaction.

Some participants cared for their mental well-being through engaging in singing and dancing. The participants’ comments relate to the perspectives of Williams, Dingle and Clift (2018:2), who indicate that participating in a choir can increase positive emotions, may reduce anxiety and often enhances social bonding between people. These impacts may be because of music aiding emotion regulation and social connectedness. According to Giannouli and Tragantzopoulou (2025:227), playing music and dancing are associated with improved cognitive health and physical well-being in tertiary students.

The study’s findings indicated that some participants relied on family members for mental health support. Close family members were available for students who needed encouragement. The findings are supported by Alharbi (2023:17), who stated that seeking social support is a common coping method among nursing students. Such support functions as a stress-protective element, as it can either reduce stress or help students respond to the challenging circumstance in a healthy way. Family members that are consulted include close relatives such as parents, children, siblings and extended family members (Yang et al. 2022:1).

According to Dias et al. (2024:327), most nursing students in their study adopted a range of coping mechanisms, including talking to friends and engaging in sports as ways to cope with mental health challenges. The above findings concur with the findings of this study, as participants indicated that they relied on their trusted friends and classmates for mental health support. Nikita et al. (2018:3) also support the research findings, indicating that peer support helps students to cope with challenging emotional and psychological experiences. It has been found that giving and receiving help respectfully in a mutual agreement between students with similar experiences contributes to their mental well-being.

The participants applauded the lecturers’ support with their academic, personal and professional challenges. They used daily encounters with the lecturers to talk to them and to arrange counselling. The lecturers were available and approachable to the extent that the students relied on them for immediate help. Stubin et al. (2024:120) support the above findings, indicating that lecturers are the first line in addressing students’ mental health problems. They are the ‘eyes and ears’ in classrooms and can deliver immediate support when students experience challenges.

Being enrolled at tertiary education institutions is, for many students, a period of transition into adulthood. According to the participants, they experienced studying to become professional nurses as very stressful, and although they were aware of the services rendered at the institutional counselling department, they preferred not to make use of it. Many nursing students are prone to developing mental health problems because of expectations from parents and lecturers and therefore require support to improve their well-being and to overcome mental health challenges (Batool, Maqsood & Malik 2023:303). Nursing students are also a highly sensitive group to do counselling with, and care should be taken to make sure that they do not perceive counselling to mean failure on their part. It has been noted that only a small proportion of students suffering from mental health problems receive counselling services (Kennedy 2025:298). In general, young people consider getting mental health counselling to be an admission of failure to deal with their own problems (Huenergarde 2018:14). The above studies concur with the findings of this study. The participants were aware of the institutional support services, but they preferred not to utilise them.

The study’s findings revealed the effectiveness of the Student Representative Council in implementing activities that promoted students’ mental health. These findings were supported by Stubin et al. (2024:122), indicating that such organisations render peer support to students that is vital for their development of resilience required to overcome stressful situations. Student organisations play critical roles in raising mental health awareness and promoting the de-stigmatisation of the use of counselling services to improve mental health (Wu et al. 2024:975). Based on the research of Kakungulu (2024:29), members of student organisations have opportunities and responsibilities to lead their peers in a variety of extracurricular activities such as drama, choir, athletics and other well-being activities. The participants of this study appreciated the support, which were arranged for students who felt overwhelmed. Seidman, Kim and Raza (2019:101) alluded that student councils have responsibilities towards students’ general well-being and the enhancement of their academic achievements.

According to Vicary et al. (2024:3), on average, students begin their tertiary education in the age bracket of 18 to 25 years, universally acknowledged as the prime vulnerability period for the development of mental health problems. Nursing students not only have to adjust to being students but also take part in patient care during work-integrated learning and therefore are exposed to the stress associated with caring for people in pain. They are obliged to manage multiple pressures related to academic achievements and the fear of making mistakes to the detriment of patients during work-integrated learning contributing to the high levels of stress that they often experience (Maharani, Dewi & Kurniyawan 2022:55). While some students cope effectively with the stress, others do not and the latter group therefore often suffer the likelihood of developing mental health problems (Wu et al. 2024:1000). The above findings from literature correlate with the input from the participants who revealed that, when nursing students do not excel academically, they often become involved in substance abuse. According to the participants, nursing students may feel so overwhelmed that they become depressed and suffer from episodes of severe anxiety. Saima et al. (2022:1) further state that experiences of severe stress in combination with maladaptive ways of dealing with stress are considered risk factors for nursing students to develop mental health illnesses.

When nursing students are exposed to long-term stress, their physical and mental health may deteriorate (Hamadi et al. 2021:3). One participant in the study indicated that nursing students may pretend that they are coping with the challenges of studying to become professional nurses but cry when others do not see them. Alkouri et al. (2025:7) alluded that nursing students who do not benefit from counselling may develop maladaptive coping strategies leading to substance abuse, which may reflect unfavourably on their overall well-being. According to the study’s participants, a lack of mental health promotion may lead to students developing ineffective coping skills, such as being involved in reckless behaviour and being addicted to substances. Based on Baluwa et al. (2025:13), professional nurses who are chemically dependent often confess that the abuse started while they were students.

The participants were concerned about the possibility that fellow students could commit suicide in order to escape challenging academic circumstances. According to Moraes et al. (2021:138), students with symptoms of depression have low lecture attendance rates, poor performance in assessments and are at risk of committing suicide. Failing to promote mental health among nursing students may lead to dire consequences, which will not only affect their academic performance but also their overall well-being. Projects to promote students’ mental health are critically needed to assist them to thrive both academically and personally.

The participants believed that promoting students’ mental health within educational institutions may enable students to excel academically and on a personal level. According to them, students need guidance to develop balanced and healthy lifestyles. Based on the perspectives of Khunsawat et al. (2025:657), enhancing students’ mental health should be a key component in tertiary education to build a generation of professional nurses who are equipped not only with academic knowledge but also with emotional resilience to thrive in an increasingly complex healthcare world.

In summary, through investment in mental health promotion, solid communities of knowledgeable people leading healthy lives are sustained. Mental health promotion empowers individuals to cope with challenges, connect with others and thrive in all spheres of their lives. Promoting mental health reduces stigma of securing optimal mental health and encourages students’ resilience when challenges are experienced.

The findings of the study highlighted that nursing students at a designated nursing institution are partially knowledgeable about mental health promotion and not all nursing students practice positive mental health mechanisms when dealing with academic and personal challenges.

### Limitations and recommendations

The study noted a need for nursing students to be enlightened about mental health promotion and activities that they can use to promote their well-being. Education institutions should not only make general support services available to help students navigate academic, financial and personal challenges. Students’ input on measures to enhance the services’ accessibility should be exploited.

Students from only one institution were involved. The participants were nursing students and could have provided answers to impress the interviewer, who was one of their lecturers, with their knowledge about mental health promotion.

## Conclusion

The study findings revealed that the participants were knowledgeable about mental health promotion, but some of them did not practice mental health promotion when dealing with academic and personal challenges.
